# Comorbidity Patterns Among Patients Diagnosed with Sialolithiasis: A Retrospective Analysis

**DOI:** 10.3390/jcm14113795

**Published:** 2025-05-28

**Authors:** Rimah Abdullah Saleem, Hatouf Husni Sukkarieh, Rana K. Alkattan, Hana M. A. Fakhoury, Ahmad Aljada, Abdulrahman Theyab, Khansa Taha Ababneh

**Affiliations:** 1 Department of Biochemistry and Molecular Medicine, College of Medicine, Alfaisal University, Riyadh 11533, Saudi Arabia; rsaleem@alfaisal.edu (R.A.S.); hhajeer@alfaisal.edu (H.M.A.F.); aaljada@alfaisal.edu (A.A.); abdalshehri@alfaisal.edu (A.T.); 2 Department of Restorative and Prosthetic Dental Sciences, College of Dentistry, King Saud bin Abdulaziz University for Health Sciences (KSAU-HS), Riyadh 11426, Saudi Arabia; dr_rkk@hotmail.com; 3 King Abdullah International Medical Research Center, Ministry of National Guard Health Affairs, Riyadh 11481, Saudi Arabia; 4 Dental Services King Abdulaziz Medical City, Ministry of the National Guard-Health Affairs, Riyadh 11426, Saudi Arabia; 5 Security Forces Hospital, Mecca 14799, Saudi Arabia; 6 Preventive Dental Science Department, College of Dentistry, King Saud bin Abdulaziz University for Health Sciences, Riyadh 11426, Saudi Arabia

**Keywords:** sialolithiasis, systemic diseases, obesity, dyslipidemia, diabetes mellitus

## Abstract

**Background/Objectives:** Sialolithiasis is a common disorder of the major salivary glands characterized by the formation of salivary stones, known as sialoliths. It is predominantly observed in patients in their fourth to sixth decades of life. Several potential factors have been associated with the development of sialolithiasis, including obesity, smoking, medication use, and several systemic diseases such as dyslipidemia, hypertension, and diabetes mellitus. Despite extensive research into the pathogenesis of sialolithiasis, it remains elusive. Therefore, this study aims to explore the characteristics of sialolithiasis among patients residing in Saudi Arabia and to detect the possible role of systemic disorders in the development of sialolithiasis. **Methods:** This study included 375 patients with a confirmed diagnosis of sialolithiasis between 1 January 2016 and 31 December 2024, at the National Guard Health Affairs centers in Saudi Arabia. Demographic information, body mass index (BMI), presence of systemic disorders (hypertension, diabetes mellitus, dyslipidemia, asthma, cancer), and the age at diagnosis of each disease were collected for data analysis. **Results:** A total of 55.5% of the patients were male, with a mean age of 39.8 and a mean BMI of 28. Dyslipidemia represented the most prevalent systemic condition (32.9%), followed by diabetes mellitus (23.5%) and hypertension (20.3%). Moreover, 74.4% and 74.2% of the patients developed hypertension and diabetes mellitus before sialolithiasis, respectively. Furthermore, dyslipidemia was associated with a higher risk profile compared with diabetes mellitus and other systemic disorders. **Conclusions:** This exploratory study indicates that the incidence pattern of sialolithiasis among both genders likely depends on lifestyle factors and other underlying systemic conditions. Elevated BMI may be a contributing characteristic, and the development of systemic diseases such as diabetes mellitus could be linked to the formation of salivary stones. Furthermore, these findings support a multifactorial pathophysiology of sialolithiasis. The aforementioned factors may play a role in the formation of salivary stones through hyposalivation, whether disease-related or medication-induced hyposalivation, potentially serving as a common mechanism.

## 1. Introduction

Sialolithiasis is a common disorder of the major salivary glands characterized by the formation of salivary stones, known as sialoliths. The estimated incidence ranges from around 1 in 15,000 to 1 in 30,000 [1]. This disorder is mainly observed in individuals between their fourth and fifth decades of life [2]. It frequently affects the submandibular glands, followed by the parotid glands, and rarely impacts the sublingual and minor glands [3,4,5]. These stones are described as calcified structures found in the ducts or within the gland, leading to the obstruction of salivary secretion and triggering pain and swelling, primarily during mealtime [2,6]. Calcium and phosphate salts are considered the main constituents of sialoliths [7,8]. The decrease in salivary flow rate, changes in pH, dehydration, and infections are thought to be associated with salivary stone formation [9].

Studies conducted prior to 1990 indicated male predominance in sialolithiasis, with a reported male-to-female ratio of 2:1 [10,11]. However, more recent studies have shown either an equal gender distribution [12] or a higher prevalence among females [13,14,15,16]. Although the mechanism of sialoliths formation remains unclear, its etiology remains elusive [7,17], several concepts have been proposed in this context, including altered concentrations of salivary ions [18], the presence of bacteria in the ducts [18,19] and the ejection of micro-calcifications of cell debris from salivary gland cells [17,20].

Various factors have been correlated with the development of sialolithiasis, including tobacco smoking, chronic antibiotic use, and diuretic medications [21,22,23]. Obesity has been implicated in inflammatory diseases such as periodontal disease, diabetes, cardiovascular disease, and fatty liver disease [24,25]. Regarding sialolithiasis, an increase in body mass index (BMI) is believed to contribute to salivary stone formation [26]. A degree of variability exists in the literature concerning the role of systemic disorders in the pathogenesis of sialolithiasis. For example, multiple studies have demonstrated an association between diabetes mellitus, hypertension, and sialolithiasis [16,27], while others have failed to indicate any association [22,28]. A similar pattern has also been observed with cholelithiasis, as one study reported a higher prevalence among sialolithiasis patients [16]; however, other studies did not find a significant association [15,21,22]. Among Saudi nationals, obesity has been highly observed in adults over 25 years of age, with higher rate in males than females [29]. According to a recent study, hypertension affects 9.2% of the Saudi population [30]. Diabetes mellitus affects 39.5 of the Saudi population. The prevalence of diabetes was 34.1% in males and 27.6% in females [31,32,33,34], and dyslipidemia ranged between 12.5% and 62% [35]. Additionally, various medications including, diuretics, antihistamines, antihypertensives, antipsychotics, antidepressants, and antiepileptic drugs have been implicated in reducing salivary flow rate, which is thought to induce sialolithiasis [9,21,22,36].

The literature lacks information regarding the prevalence and role of comorbidities in the progression of salivary stone formation in Saudi Arabia. Therefore, this study aims to explore the characteristics of sialolithiasis among Saudi Arabian patients and identify the potential role of systemic disorders in its development.

## 2. Methodology

### 2.1. Methods

This study complies with the Strengthening the Reporting of Observational Studies in Epidemiology (STROBE) reporting guideline for cohort studies. All procedures were reviewed and approved by the National Guard Health Affairs (NGHA) institutional review board (IRB) under the reference number 00000101525. The requirement for informed consent was waived due to the retrospective nature of the electronic health record (EHR) data utilized.

### 2.2. Study Setting

This study utilized retrospective patient data from the electronic health records (EHRs) of various NGHA centers in Saudi Arabia. The participating centers include a combination of general and specialty care facilities, encompassing both outpatient and hospital-based clinics. Data were de-identified and aggregated from multiple institutions with standardized health information systems.

### 2.3. Study Period

Patient visits and clinical encounters were extracted between 1 January 2016 and 31 December 2024. This timeframe was chosen to capture the full spectrum of comorbidity diagnoses surrounding the diagnosis of sialolithiasis.

### 2.4. Participants

This study included 375 patients with a confirmed diagnosis of sialolithiasis during the study period. Patients were identified using International Classification of Diseases, 10th Revision (ICD-10) codes specific to sialolithiasis (K11.5). Inclusion criteria included records of the first diagnosis date for sialolithiasis, documentation of key demographics (gender, age, and marital status), and the availability of comorbidity status (hypertension, diabetes mellitus, dyslipidemia, asthma, and cancer). Patients were excluded if their diagnosis records were incomplete, if they had missing date-of-birth information, or if medication or comorbidity variables were unavailable.

### 2.5. Data Collection

All included data were extracted from institutionally maintained EHR systems (BestCare). Each patient record contained time-stamped entries for diagnoses, documentation of comorbidities, and clinical metadata. Comorbidity dates were tracked for the first documentation and were used to determine their timing relative to the onset of sialolithiasis.

### 2.6. Patient Characteristics

Patient characteristics included gender, age at diagnosis, current age, marital status, BMI, and five systemic disorders: dyslipidemia, diabetes mellitus, hypertension, asthma, and cancer.

### 2.7. Statistical Analysis

Descriptive analyses characterized the patient population by age, gender, marital status, and comorbidities. Comorbidity frequencies were plotted using JAMA-style bar plots, which displayed percentages and confidence intervals. Temporal relationships between the diagnosis of each comorbidity and sialolithiasis were assessed by calculating the date difference (in days) and classifying patients as “diagnosed before” or “after” the primary condition. The binomial test was used to evaluate whether a greater number of patients were diagnosed before or after the onset of sialolithiasis, while violin plots were employed to present the timing distribution for each comorbidity. For regression analysis, a generalized linear model (GLM) was applied to assess predictors of earlier diagnosis, using the first diagnosis date as the outcome. Additionally, gender, marital status, and number of comorbidities were included as predictors.

Furthermore, quantile regression was employed to account for non-normal residuals. Patients were classified into high-, moderate-, and low-risk phenotypes to explore risk stratification using k-means clustering based on their comorbidities. Principal component analysis (PCA) was performed to visualize clustering patterns in a reduced-dimensional space, and summary tables described each cluster’s comorbidity profiles. Statistical significance was set at *p* < 0.05 (two-sided). All analyses were performed using Python v3.11 with packages including pandas, scipy, statsmodels, seaborn, matplotlib, and sklearn. Given the inherent complexity and variability of HER data, we employed a systematic approach to address missingness. Variables were first assessed to determine the extent and patterns of missing data. Variables with more than 20% missingness, such as BMI, were carefully evaluated for clinical relevance; those deemed non-essential or non-informative were excluded from multivariable modeling to reduce bias and preserve statistical power. For variables retained in the analysis, we assumed that data were missing at random (MAR) based on observed covariates. We applied multiple imputation using chained equations (MICE) to impute missing values, incorporating all relevant predictors and outcome variables to maximize the plausibility of the MAR assumption [37]. For sensitivity analysis, results from complete-case analyses were compared with those from imputed models to assess the robustness of the findings. No meaningful differences in the direction or magnitude of associations were observed, supporting the validity of the imputation approach.

Generative AI tools were used to aid in grammar correction and language enhancement during the manuscript’s preparation. However, none of these tools were utilized in the study’s design, data collection, analysis, or interpretation of the results.

## 3. Results

Among the 375 patients diagnosed with sialolithiasis, the majority were male (208 patients; 55.5%), while female patients accounted for 44.5%. Most patients were married (60.3%), 30.4% were single, and smaller proportions were widowed (4.0%), divorced (2.1%), or had missing marital status data (2.9%). Patient diagnoses were relatively evenly distributed from 2016 to 2024, with a peak in 2019 (15%), and diagnosis frequencies slightly declined in later years (Table 1).

The mean age at the time of data extraction was 44.7 years (SD, 17.6), while the mean age at diagnosis was 39.8 years (SD, 17.4). The average age at diagnosis was 38 years, with an interquartile range (IQR) of 27 to 53 years, indicating a relatively broad distribution of ages among affected patients. Among patients with available BMI data (*n* = 216), the mean BMI was 28.0 (SD, 10.8), which falls within the overweight range, accompanied by a median BMI of 27.3 (Table 2). The comorbidity count recorded in 187 patients had a mean of 0.96 (SD, 1.19), and a median ranging from 0.0 to 5.0, suggesting that while many patients had no comorbidities, a subset exhibited a significant burden of multimorbidity. The most common comorbid condition was dyslipidemia (123 patients; 32.8%), followed by diabetes mellitus (88 patients; 23.5%) and hypertension (76 patients; 20.3%). Asthma and cancer were less prevalent, reported in 13.9% and 8.8% of the cohort, respectively (Table 3).

**Figure 1:**
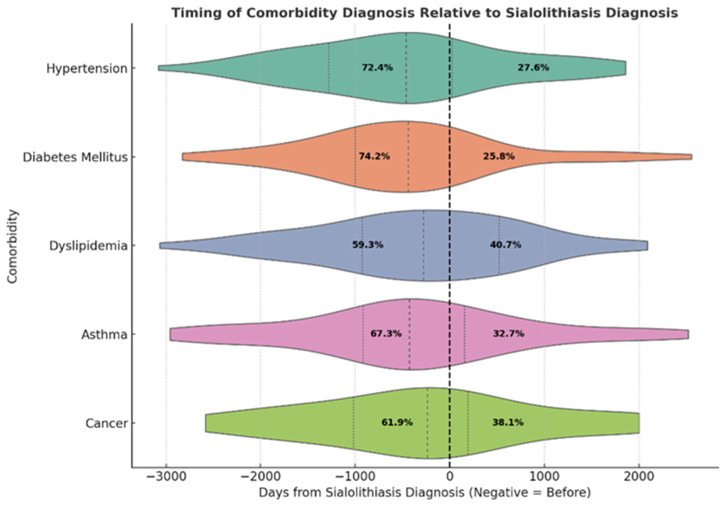
**Timing of comorbidity diagnosis relative to sialolithiasis diagnosis.** This ridge-style violin plot displays the distribution of diagnosis dates for five major comorbidities—hypertension, diabetes mellitus, dyslipidemia, asthma, and cancer—relative to the date of the first sialolithiasis diagnosis (denoted by the vertical dashed line at 0). The x-axis represents the number of days from the sialolithiasis diagnosis, where negative values indicate comorbidities diagnosed prior to the primary condition, and positive values indicate diagnosis afterward. Each violin shape visualizes the density and interquartile spread of diagnosis intervals for that condition. Inside each plot, the percentages reflect the proportion of patients diagnosed before or after the sialolithiasis diagnosis date. These distributions reveal that most comorbidities are commonly diagnosed prior to sialolithiasis, particularly hypertension and diabetes, suggesting a potentially shared risk profile or delayed diagnosis of the salivary gland condition.

Among patients with complete data on the date of diagnosis, the proportion of those diagnosed with comorbidities before their sialolithiasis diagnosis varied across conditions. A significantly more significant proportion of patients were diagnosed with hypertension (77.6%) (*p* < 0.001) and diabetes before (70.7%) (*p* < 0.001) their sialolithiasis diagnosis, while the timing for dyslipidemia (58.5%) (*p* = 0.137), asthma (48.0%) (*p* = 0.763), and cancer (45.8%) (*p* = 0.611) did not significantly differ from random (50/50) distribution (Figure 1).

**Figure 2:**
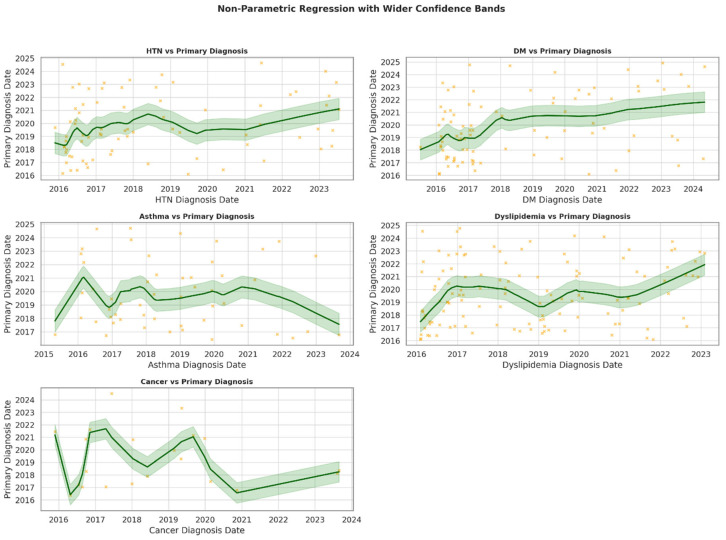
**Association between comorbidity diagnosis dates and the onset of sialolithiasis.** This panel illustrates smoothed regression trends between the first diagnosis dates of comorbidities and the onset of sialolithiasis. Each subplot displays a non-parametric locally weighted scatterplot smoothing (LOWESS) curve (dark green) along with shaded 95% confidence intervals (light green, ±200 days) and patient-level data (orange dots). The linear regression *p* value is included in each panel to assess the significance of the temporal association. HTN: hypertension, DM: diabetes mellitus.

These data also show significant positive associations between earlier diagnoses of hypertension (*p* = 0.049) and diabetes mellitus (*p* = 0.001) and the timing of salivary gland stone diagnosis, suggesting that these conditions may precede or contribute to earlier recognition of sialolithiasis. Conversely, asthma (*p* = 0.693), cancer (*p* = 0.687), and dyslipidemia (*p* = 0.104) showed no statistically significant trends, although dyslipidemia approached borderline significance (Figure 2).

**Figure 3:**
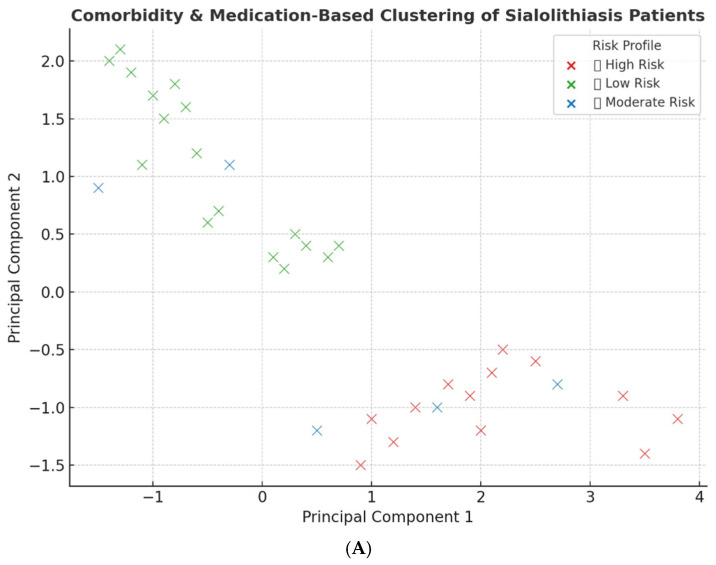

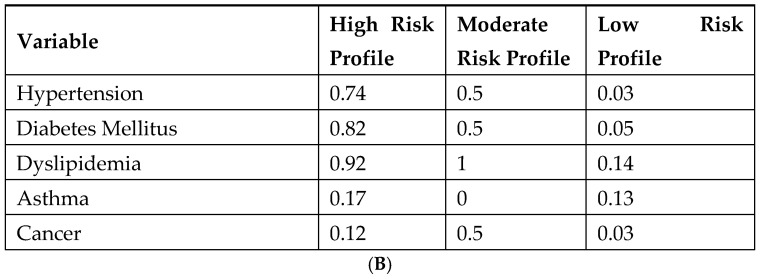
Comorbidity-based clustering of sialolithiasis. (**A**) The principal component analysis identified three clusters: high-, moderate-, and low-risk profiles. (**B**) The table represents the burden of chronic disease based on the risk profile.

Using principal component analysis (PCA) on comorbidity data, three distinct patient clusters were identified, corresponding to high-, moderate-, and low-risk profiles (Figure 3A). Each cluster demonstrated unique patterns of disease burden management. The chronic diseases with the highest risk profile were 74% hypertension, 82% diabetes mellitus, and 92% dyslipidemia. Interestingly, dyslipidemia presented a 100% moderate risk profile while hypertension, diabetes mellitus, dyslipidemia, and cancer each presented in 50% of cases. Furthermore, the low-risk cluster demonstrated a comorbidity burden of 3% for hypertension, 5% for diabetes mellitus, and 14% for dyslipidemia (Figure 3B).

## 4. Discussion

This study aimed to explore the prevalence of sialolithiasis among Saudi patients and determine the potential factors associated with the development of this disorder, specifically diabetes mellitus, hypertension, dyslipidemia, asthma, and cancer. It is widely recognized that reduced saliva flow contributes to the development of sialolithiasis [20]. Changes in the pH have also been linked to the formation of salivary stones influenced by several factors such as increased calcium content and crystalloid solubility impairment [5]. Nevertheless, many studies have explored the potential factors related to the pathophysiology of sialolithiasis, which remains a complex issue. Herein, we demonstrated the characteristics of patients suffering from sialolithiasis and determined the impact of systemic diseases on the development of this condition among Saudi patients.

Although sialolithiasis is a common salivary gland disorder [38]; its incidence pattern varies across different populations. For instance, our findings revealed a higher number of sialolithiasis cases compared to a study conducted in the Netherlands, even though our data were collected over a shorter span of nine years, versus their twelve-year study period [22]. Another research study recorded 40 cases within one year, which aligns closely with our observation during the same timeframe [7]. Geographic and socioeconomic factors such as place of residence, diet, smoking habits, drinking water composition, and consumption levels have been suggested to influence salivary stone formation [13,26]. These elements have been thoroughly studied within the Saudi population. For instance, reports indicate that 21.4% of Saudi adults smoke [39], with a higher prevalence among men than women [40]. Smoking is believed to play a role in sialolithiasis by lowering salivary flow and diminishing antimicrobial activity [23,41]. Age has been linked to xerostomia, and medical conditions such as diabetes mellitus have also been associated with its development [42,43]. A study in Saudi Arabia revealed that 38.7% of people consume less than 1 L of water daily, whereas fewer exceed this amount [44] likely due to exposure to the hot and humid climate and the increased fluid demands from exercise [45]. In this context, it has been proposed that drinking water rich in calcium increases salivary calcium levels, potentially contributing to the development of salivary stones [7,41].

Several studies have reported an equal incidence rate for both genders [46,47]. Interestingly we noted a higher incidence of sialolithiasis in males compared to females, which corresponds with earlier findings prior to 1990 [10,11,48], and more recent studies [47,49]. Conversely, others reported a higher prevalence of sialolithiasis among females [13,26]. This finding was unexpected, as women typically exhibit a lower salivary flow rates than men [50,51], a factor thought to contribute to sialolith formation. These findings emphasizes that lifestyle, systemic-related disorders, or other underlying factors may be responsible for the progression of sialolithiasis.

Research indicates that sialolithiasis affects individuals between 30 and 60 years of age, with an average age of 45 [5,16,52]. This aligns with our findings, although the mean age at diagnosis in our cohort was slightly lower (at 39.8). Recent population trend analyses suggest that, because this disorder is associated with older individuals, the decline in sialolithiasis cases after 2021 may be attributed to the current Saudi population being predominantly under the age of 34 [53]. In addition, it implies that lifestyle behaviors probably have a more significant influence than advancing age.

The BMI is a measurement tool for determining body fat composition, categorizing individuals as underweight, normal, overweight, or obese. A BMI of 25–29 is classified as overweight, while a BMI above this range is deemed obese [54]. Recent studies have identified elevated BMI as risk factor for sialolith formation [26]. In our study, the average BMI of the patients was 28, placing them in the overweight category and approaching the threshold for obesity. Previous findings have reported that a BMI over 30 in men and over 25 in women is linked to periodontitis [24]. Although Jin et al. found no link between obesity and sialolithiasis, it has been suggested that obesity may lead to chronic inflammation, which could promote salivary stone formation [47]. In addition, an elevated BMI is associated with the development of sialolithiasis, presumably due to poor oral health [55]. However, further investigation is required to confirm the association between BMI and sialolithiasis.

It has been suggested that sialolithiasis may arise from dehydration, reduced fluid intake, radiotherapy-induced xerostomia (dry mouth sensation), and certain medications [56]. We hypothesized that systemic disorders such as dyslipidemia, diabetes mellitus, hypertension, asthma, and cancer may play a role in sialolithiasis. Although the prevalence of these diseases was relatively low compared to the overall study population, a notable association was identified between sialolithiasis and systemic diseases; particularly dyslipidemia, diabetes mellitus, and hypertension. To our knowledge, this is the first study to determine such an association within the Saudi population and the first study to link the onset of systemic diseases to sialolithiasis.

In this context, Jonas et al. found an association between dyslipidemia and the formation of salivary stones [16]. Obesity has been identified as a key factor in the progression of metabolic diseases, including hypertension, insulin resistance, and dyslipidemia [57]. Dyslipidemia is defined by an abnormal lipid profile [58], which affects approximately 12.5–62% of the Saudi population [35,59]. Previous studies have investigated the role of metabolic disorders in sialolithiasis through BMI and lipid levels [26,47]. Approximately 39.8% of Saudi patients suffer from metabolic disorders, characterized by reduced high-density lipoprotein (HDL), increased low-density lipoprotein (LDL), and elevated BMI [60,61]. These levels may be attributed to unhealthy eating habits prevalence in the Saudi population, which are influenced by excessive appetite and frequent fast-food consumption [62]. Abnormal lipid levels have been associated with reduced the salivary flow rates [63,64], which can lead to sialolith formation through the precipitation of calcium salts [16]. Although the inorganic materials comprise most of the sialolith composition, whether from the submandibular or parotid glands [65], phospholipids, cholesterol, cholesterol esters, and fatty acids have also been detected [66,67,68].

It has been reported that medications may lead to the formation of salivary stones [7,16].

For example, patients with hypertension are often treated with a variety of medications, including beta blockers, angiotensin-converting enzyme inhibitors, and diuretics. These medications can induce hyposalivation and modify salivary composition through different mechanisms, such as interacting with salivary gland alpha-2 adrenergic receptors and altering calcium homeostasis [69]. This reaction may promote the formation of sialoliths by raising the levels of calcium and other minerals in saliva while reducing saliva production [69,70]. Moreover, Thomas et al. have highlighted the link between anti-asthmatic drugs and oral health [71]. Certain powder inhalers contain sugar, which reduces in saliva secretion and a decrease in salivary pH. In addition, the use of beta-2 adrenergic agonists by asthma patients may induce similar effects on saliva and increase the proliferation of cariogenic microorganisms [72,73]. It is well established that patients with head and neck cancer patients are susceptible to xerostomia due to radiotherapy [74]. Patients with other types of malignancies may also experience hyposalivation after cancer treatments, although this occurs to a lesser degree of severity, as the adverse effects of chemotherapy and immunotherapy are temporary [75]. However, these suggestions should be further explored to confirm the role of the long-term use of medications for chronic diseases and cancer in sialolith formation.

Aside from hypertension and cancer, the clinical characteristics of diabetes mellitus are believed to be responsible for hyposalivation [76]. This condition might result from damage to the gland parenchyma, alterations in the microcirculation to the salivary glands, dehydration, and disturbances in glycemic control [77,78].

## 5. Limitations

This observational study sheds light on the prevalence and potential role of systemic diseases in sialolithiasis; however, the sample size is relatively small given the low incidence rate of the condition (1 in 15,000 to 1 in 30,000). Further large-scale studies involving broader patient populations across diverse healthcare institutions are recommended to more comprehensively understand the pathophysiology and risk factors associated with sialolithiasis at a national level. In addition, this preliminary analysis focused on the characteristics of patients with sialolithiasis and the potential impacts of systemic disorders on the progression of this disorder. However, it would be interesting to include a control group, which could help to better evaluate the significance of each systemic condition on the development of sialolithiasis.

## 6. Conclusions

These observations reported in this study highlight the value of evaluating the systemic disease history of individuals to identify those at higher risk of early-onset sialolithiasis. Our findings suggest that sialolithiasis development is influenced by biological mechanisms and behaviors associated with systemic conditions. Furthermore, the etiology appears to be multifactorial rather than attributable to a single factor, primarily involving salivary gland hypofunction resulting from disease pathology or the side effects of medications used to treat systemic disorders. Considering these insights, we conclude that prolonged exposure to multiple risk factors likely contributes to the pathogenesis of sialolithiasis. Future research efforts will include prospective metabolic and proteomic analyses of saliva samples from patients with sialolithiasis, both with and without systemic conditions, in order to provide deeper insights into the underlying pathophysiology. The presented findings also highlight the need to identify associations between systemic diseases (e.g., hypertension) and medication intake with the formation of sialoliths, as well as to more broadly investigate the incidence pattern of salivary stones among hypertensive patients.

## Figures and Tables

**Table 1:** Demographic characteristics and frequency of diagnosis of sialolithiasis between 2016 and 2024.

Variable	Category	N (%) ^1^
Gender	Male	208 (55.5%)
	Female	167 (44.5%)
Marital Status	Married	226 (60.3%)
	Single	114 (30.4%)
	Widowed	15 (4.0%)
	Unknown	12 (2.9%)
	Divorced	8 (2.1%)
Year		
	2016	43 (11.5%)
	2017	49 (13.1%)
	2018	48 (12.8%)
	2019	56 (15%)
	2020	44 (11.8%)
	2021	40 (10.7%)
	2022	42 (11.2%)
	2023	27 (7.2%)
	2024	25 (6.7%)

^1^ N(%) = sample size and percentage.

**Table 2:** Summary of current age, age at diagnosis, body mass index (BMI), and comorbidity count among patients with sialolithiasis. Values are presented as mean ± standard deviation (SD) and median with interquartile range (IQR). N indicates the sample size for each variable.

Variable	N ^1^	Mean Â ± SD	Median [IQR]
**Current Age**	375	44.7 ± 17.6	43.0 [31.0–58.0]
**Age at Diagnosis**	373	39.8 ± 17.4	38.0 [27.0–53.0]
**BMI**	216	28.0 ± 10.8	27.3 [22.6–31.4]
**Comorbidity count**	187	0.96 ± 1.19	5.0 [0.0–5.0]

^1^ N = sample size.

**Table 3:** Distribution of systemic conditions among patients with sialolithiasis. Data are presented as number of patients (N) and corresponding percentages (%). N (%) = sample size and proportion within each category. Total sample size = 375.

Variable	Category	N (%) ^1^
Hypertension	No	299 (79.7%)
	Yes	76 (20.3%)
Diabetes Mellitus	No	287 (76.5%)
	Yes	88 (23.5%)
Dyslipidemia	No	252 (67.2%)
	Yes	123 (32.8%)
Asthma	No	323 (86.1%)
	Yes	52 (13.9%)
Cancer	No	342 (91.8%)
	Yes	33 (8.8%)

Total N 375. ^1^ N(%) = sample size and percentage.

## Data Availability

The data supporting this study’s findings are available from the corresponding author upon reasonable request.
